# Growth, allergen profile and microbiome studies in *Dermatophagoides pteronyssinus* cultures

**DOI:** 10.1038/s41598-023-37045-9

**Published:** 2023-06-30

**Authors:** D. Calzada, L. Martín-López, Jerónimo Carnés

**Affiliations:** R&D Unit, Allergy & Immunology, LETI Pharma S.L.U., Calle del Sol nº 5, Tres Cantos, 28760 Madrid, Spain

**Keywords:** Microbial communities, Microbiome, Immunology, Microbiology, Diseases

## Abstract

Mites are mass-cultured to manufacture allergen extracts for allergy diagnostics and therapeutic treatment. This study focused on characterizing the growth, the allergen profile, and the microbiome of *Dermatophagoides pteronyssinus* cultures. Mite population, protein profile, total protein content and major allergen levels (Der p 1, Der p 2, Der p 23) were monitored at different times of three independent cultures. The allergenicity was studied by immunoblot using a pool of sera from allergic patients. Mite microbiome was characterized by sequencing the 16S rRNA gene from 600 adult mites from the last day of the culture. Endotoxin content was also analyzed. The cultures had a fast and unrelenting evolution. Mite density, total protein content, major allergen levels and the allergenicity were increased progressively during the cultures. Regarding the microbiome studies, the results confirm the presence of non-pathogenic bacteria, being firmicutes and actinobacteria the most common bacterial taxa, with a very low content of Gram-negative bacteria and endotoxin content. The allergenicity and levels of the main allergens in the mite cultures are objective methods useful to monitor the mite culture that help to produce standardized allergen extracts. The high presence of Gram-positive bacteria found limits the possibility for vaccine contamination by bacterial endotoxins.

## Introduction

*Dermatophagoides pteronyssinus* is one of the most predominant species of house dust mites (HDMs) worldwide^1,2^. It is particularly abundant in mattresses, upholstery, and sofas. To date, 32 allergens of *D. pteronyssinus* have been identified. This wide range of allergens cause allergic rhinoconjunctivitis and asthma and contribute to other allergic skin diseases in genetically predisposed individuals. Specifically, Der p 1, Der p 2 and Der p 23 are considered the major allergens due to their serodominance and their clinical relevance^3,4^. Therefore, an exhaustive characterization and standardization of mite allergen extracts are essential for allergy diagnosis and treatment of mite allergy^5^.

The manufacturing of allergenic extracts implies the culture of mites in large quantities. Mites reproduce sexually, and their development includes the following stages: egg, prelarva (inactive and develops inside the egg), larva, protonymph, tritonymph, and adults^6^. HDM cultures show different growth patterns at different times during culture development: a latency phase characterized by a slow increase in the mite population, and exponential phase resulting in a maximum population of mites, and finally a death phase of the culture^7^. Controlled conditions are required for a suitable growth in an appropriate culture media. Specifically, metabolism and reproductive rate are affected by temperature and relative humidity. Optimal temperature (20–25 °C) maintains culture mites in an adequate reproductive rate and relative humidity levels above the 75% allows mite to maintain water equilibrium, necessary because mites are critically dependent on ambient water vapor to absorb water^3^. Moreover, differences in allergenic profile have been observed in allergen extracts from different culture growth phases^8,9^ and the presence and quantity of allergens could vary during the period of the mite population. Therefore, deciding when to harvest are essential to obtain the most suitable allergenic composition of mite allergen extracts^1^. The exponential growth stage is the most commonly employed for extract production^5^. However, current methods control the growth manually, and live mites are usually count using a dissecting microscope, which required time and qualified staff.

In addition, laboratories maintain mite cultures as a source of material to use for research purposes increasing the knowledge about this allergenic source. In this sense, some studies have suggested that the microbiome composition can affect the host´s biology, that includes allergen expression and the production of bacterial endotoxins that could potentially explaining the variability in allergens in mite extracts^9^. Specifically, internal mite microbiota could interact with the major allergens Der p 1, Der p 2 and Der p 23 due to their biological functions and their presence in mite gut^5^. Moreover, recent studies had focused on how the temporal changes in house dust mite microbial communities vary at different stages of mite culture development^5,10^. Hypothetically, the death phase of mite culture could be caused by acaropathogenic bacteria that were poisoning the mites mainly because the presence of dead bodies of mites, feces and diet debris^11^. However, the biological relationships between mites and bacteria are still poor understood and the factors that affect the dynamics of microbial communities are not elucidated^10^. In this sense, more studies that clarify the implications of microbiome in mite cultures are needed. Currently, different methods for research the microbiota have undergone several improvements. The techniques have evolved from traditional bacterial cultures to of new advances in next generation sequencing. This method allows knowing the whole composition of microbiota instead of the use of convectional bacterial isolation and culture methods where only few genera could be determined^12^.

The present study focused on characterizing, in each stage of culture, the protein content and allergen profile as alternative methods to evaluate the growth of mite cultures. Additionally, the whole microbiome of *Dermatophagoides pteronyssinus* cultures was sequenced and compared among the three different cultures at the end of the culture to elucidate the importance of bacteria in the growth of mites and allergen expression.

## Results

### Protein content and major allergens levels correlate with mite culture growth

**Figure 1:**
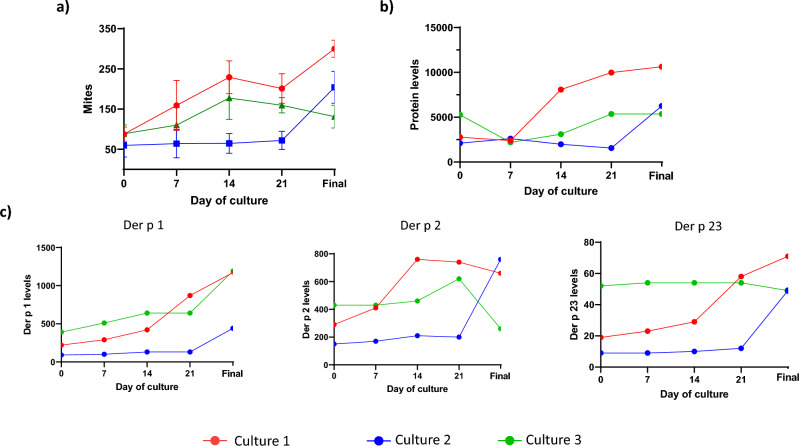
Changes in (**a**) population density (nº mites/mg culture), (**b**) protein content (ng protein/mg culture) and (**c**) major allergen levels (ng allergen/mg culture) during culture growth of *Dermatophagoides pteronyssinus*.

Mite cultures were grown in absence of contaminant during the whole period, the temperature and humidity was maintained in time, and non-significant deviations were observed. Regarding mite population, Fig. 1a shows the curve of growth of each culture. Culture number 1 had a fast and unrelenting evolution and finished with the highest concentration of mites (300 mites/mg culture). Culture 2 had a low growth index, the latency period was large but in the last days of the culture, mites grew up exponentially. Culture 3 had a discontinuous rate of growth with a high variability during the 30 days of culture, reason for which the culture was finished.

Differences in protein content and allergen production have been observed in mites from different culture growth phases. Regarding total protein levels, the curve profile of the protein levels was similar to the mite growth curves. Culture 1 was the batch with the highest levels of extractable protein at the final day of culture (10,630 ng protein/mg culture) and culture 2 increased the levels of the total protein only during the last days of culture, Fig. 1b. According to major allergens, final levels of Der p 1 were similar in culture 1 and 3 (1170 and 1190 ng/mg culture, respectively). The final levels of Der p 1 in culture 2 were 440 ng Der p 1/mg culture (Fig. 1c). However, this last culture had the highest levels of Der p 2 (760 ng/mg culture) whereas the lowest levels measured was in culture 3 (260 ng/mg culture). Der p 23 was the less abundant allergen. The highest levels measured were in the final stage of culture 1 (71 ng/mg culture). The final levels in cultures 2 and 3 were 49 ng/mg culture.

**Figure 2:**
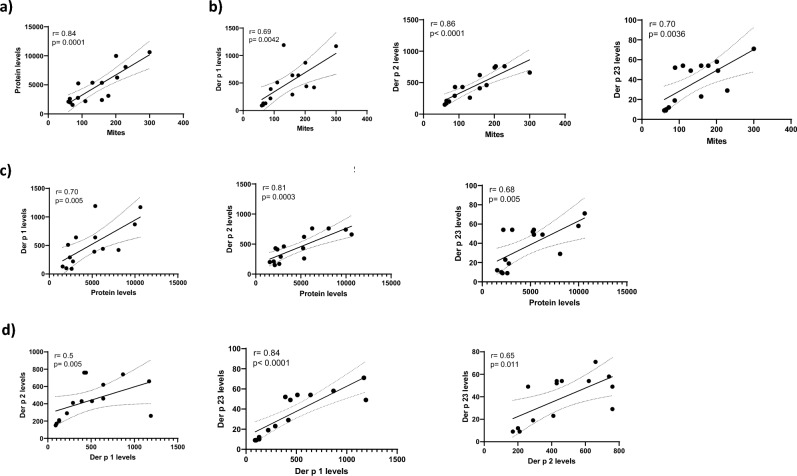
Correlations among the different variables measured in the cultures. (**a**) Correlation between the mite population (nº mites/mg culture) and total protein content (ng protein/mg culture). (**b**) Correlations between mite population and the quantities of each of the three major allergens (ng allergen/mg culture). (**c**) Correlations between the total protein content and the quantities of each of the three major allergens. (**d**) Correlations between the levels of each pair of major allergens. Pearson *r* and *p*-values are shown in each graph.

Significant correlations were observed among all the parameters tested (Fig. 2). Regarding the analysis among major allergen abundance and mite density, Der p 2 was the allergen that presented the highest correlation (r = 0.86, p < 0.0001), Fig. 2b. Der p 2 levels correlates very good with respect to total protein content (r = 0.81, p = 0.0003), Fig. 2c. Correlations among major allergens also presented good correlations, being Der p 1 and Der p 23 the allergens with the best one (r = 0.84; p < 0.0001), Fig. 2d. However, the two most abundant allergens, Der p 1 and Der p 2, presented the lowest correlation (r = 0.5, p = 0.005).

### Protein and allergenic profile of mite culture increases with the culture growth

**Figure 3:**
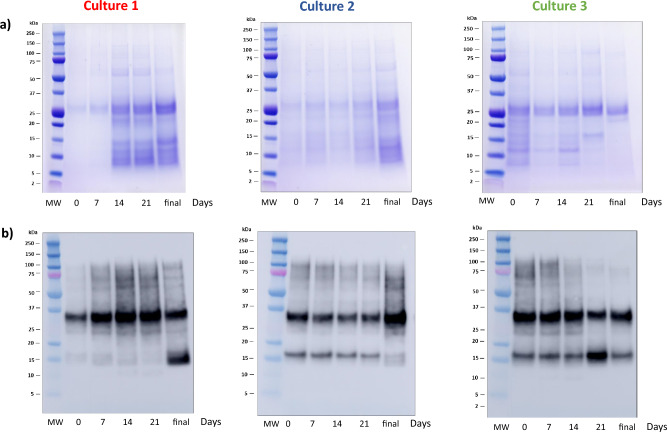
(**a**) Protein and (**b**) allergen profile during the *D. pteronyssinus* culture. The maximum volume (40 μl) allowed by the system was loaded in each lane. Gels and western blots are original images. Time exposure of blot was 1.5 s.

Protein profile of different cultures is shown in Fig. 3a. Culture 1 and culture 2 increased the number and the intensity of bands in the SDS-PAGE during mite culture development, being the most intense bands at the end of the culture. In the case of culture 3, the band intensities decreased along the time, this fact was also observed for the total protein content in the Bradford analysis and for Der 1 and Der p 23 quantification. The variability in the allergen profile of the samples recruited during the culture was analysed by western blot using a pool of sera from patients allergic to *D. pteronyssinus*. Figure 3b summarizes the results obtained in the three batches. The recognition of allergenic protein was more intense in the last stage of cultures for culture 1 and 2, whereas in culture 3, the recognition intensity decreased during the time, as it was also observed by the SDS-PAGE.

### The microbiome of *D. pteronyssinus* compromised various bacterial taxa and Gram-positive bacteria community

**Figure 4:**
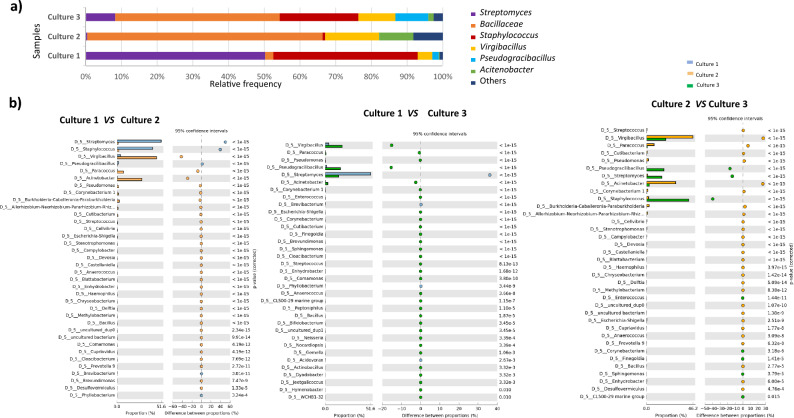
(**a**) Phylogenetic assignment of bacterial 16S rRNA gene sequences cloned from mite whole bodies homogenates in *D. pteronyssinus* samples. (**b**) Comparative statistical analysis among relative frequency of genus in the three mite cultures.

The internal mite bacteria community was largely composed of *Streptomyces*, genera of Bacillaceae family; *Staphylococcus, Virgibacillus, Pseudogracibacillus,* and *Acinetobacter* (Fig. 4a)*.* All of them are Gram-positive bacteria.

Culture 1 presented the highest relative frequency of *Streptomyces* (50%) and *Staphyloccus* (40%) but the lowest percentage of other Bacillaceae (2.3%) and *Virgibacillus* (4.1%). This genus was most abundant in culture 2 (15.15%) and 3 (10.36%) with statistically significant differences compared to culture 1. The presence of *Streptomyces* was very low in culture 2 and 3 (0.43%, 8.26%, respectively) and with statistically significance differences among culture 1. Moreover, the family Bacillaceae was the family with the highest relative frequency in the last two cultures (65.91% in case of culture 2 and 46.1% in culture 3). All statistically significant differences observed among the cultures are indicated in Fig. 4b.

Regarding the endotoxins content, the three cultures presented low levels at the final stage of the culture, Culture 1: 9.39 UE/ml, culture 2: < 1 UE/ml and 9.9 UE/ml and culture 3: 2.63 UE/ml.

## Discussion

Growing mites required to maintain cultures in proper conditions to guarantee and harmonize the raw material to produce allergen extracts. In that sense, the use of new molecular and genomic approaches could offer relevant advantages to standardize and measure the quality of live-mite growth profile. The dynamic of *D. pteronyssinus* culture was extensively analyzed in this article and revealed how the mite growth could affect the allergen production, which is to the best of our knowledge, the first time that the three major allergens are monitored along the culture. Furthermore, the mite microbiome study provides an extra-value to elucidate the importance of the bacteria presence and how can affect to the growth and allergen profiles.

Two of the three mite cultures grew properly with specific growth patterns at different times during culture development: a latency phase characterized by a slow increase in the mite population, and exponential phase resulting in a maximum population of mites^7,10^. However, the third culture presented a discontinuous rate of growth with a high variability during the 30 days of culture. The results of protein analysis also indicates that fact. Mite populations were proportional with the levels of protein measured in the extracted solutions, that can also be visualized in the SDS-PAGE. Furthermore, the intensity and number of recognition bands are directly related with the protein levels detected in the western blot.

Regarding the study of main allergens, the most abundant allergen was Der p 2, which is associated with mite bodies but can also be found in fecal pellets, and Der p 1, that is abundant in feces, both are digestive enzymes with protease activities. Der p 23 is mainly presented in the outer membrane of mite feces but in lower concentration, as was previously described^13,14^. Despite the low concentrations in natural environment, it is considered a potent allergen, with a positive sensitisation rate of 75% in allergic patients, with a high impact in children with persistent moderate-to-severe asthma^4,15^.

According to the correlation among mite population and major allergens, the study indicated that levels of Der p 1, Der p 2 and Der p 23 could be a useful, quick, and objective approach to monitor the culture. This utility was also demonstrated to analyze the levels of mite allergens that patients are directly exposed to, using house dust samples^4^. In the present study, the correlation between Der p 2 levels and mite population was the highest (r = 0.86), mainly because this allergen is associated to mite bodies. Regarding the correlation among allergens, Der p 1 and Der p 23 presented the highest correlation (r = 0.84), the allergens that are presented mainly in feces.

On the other hand, we have characterized the bacterial communities in the mite cultures. Previous studies described that the microbiome of mite cultured plays a critical role in the mite growth^12^, bacteria enter to the gut by ingested diets, produce exoenzymes that modify the environment in which mites are growing and their proteases could digest difficult nutrients. Other studies have demonstrated that different species or even different population of the same allergen-producing mites differ in microbiome composition and that this can be correlated with different expression of known allergens. This fact was demonstrated in the mite specie *Tyrophagus putrescentiae* where the expression of groups of allergens 4, 7, 10, 11, 13, 20 and 36 was correlated with the presence of intracellular bacteria as *Wolbachia* and *Blarrabacterium*-like bacteria^5,16^.

Regarding our mite cultures, non-pathogen bacteria was found in *D. pteronyssinus* microbiota, in line with previous studies^5^. Similarly, Firmicutes and Actinobacteria were the only *phylum* that we have found. The presence of these bacteria may also indicate external rumen feeding, that participate in the predigestion of mite food by enzymes that prehydrolyze structural polysaccharides to oligosaccharides^17^. Both bacterial taxa occur frequently in stored-product mites. Moreover, Actinobacteria are naturally presented in human skin, hairs, and nails and may represent a food source of wild HDMs^7^. The different microbiome profile obtained in the three cultures could explain the different growth profile found. Interestingly, the sample of culture 1 had the highest density of mites and the lowest abundance of *Virgibacillus*, the differences in percentage of abundance of this genus was statistically significant compared to cultures 2 and 3. This fact is in accordance with other study that the bacteria from the genus *Virgibacillus* was negatively correlated with mite density in a *D. pteronyssinus* culture^10^. The high presence of *Staphylococcus* and *Bacillus* are in line with other article and its presence in other industrial and laboratory cultures of *T. putrescentiae, D. pteronyssinus and L. destructor*^16–18^.

The presence of endotoxin producing Gram-negative bacteria is always a potential concern, suggesting that mite-associated microbial communities need to be continuously monitored^5^. The levels of endotoxin are associated with the endosymbiont bacteria presented in mites^19^. In our study we observed a high proportion of Gram-positive bacteria (Actinobacteria and Firmicutes), as it was demonstrated in other studies, not only in the case of *D. pteronyssinus* and *D. farinae* cultures^5,8,10,17^, also for other domestic mites as *G. domesticus*, *L. destructor* and *T. putrescentiae*^17,20^. In those cases, the presence of Firmicutes was more than 75% in *G. domesticus*, *L. destructor* and the 50% in *T. putrescentiae* culture in which the presence of Actinobacteria was higher. In contrast, the presence of Gram-negative bacteria were more abundant in *B. tropicalis* cultures than in other house dust mite cultures, being the presence of *Erwinia* genus (Proteobacteria) extremely high (99%), this enteric bacterium formed the 99% of bacteriome^17^. The high presence of Gram-positive bacteria in our study are consistent to the results of endotoxins analysis, where low levels were found in the three culture samples. These results are very relevant because the absence of endotoxins avoid the immunotherapeutic extract contamination^10^.

Mite culture and purification process are the main factors responsible for the final composition of manufacturing the allergen extract. An effort to know the kinetics of growth of mite culture and how the environment could modify the growth pattern is essential. Our study has demonstrated that the protein content and allergen profile are objective methods useful to monitor the culture that help to produce mite high-quality allergen extracts. Moreover, the internal microbiome is an interesting environmental factor that can influence in the culture development; however, more studies are required to completely understand the actual implications in the allergen production.

## Methods

### *Dermatophagoides pteronyssinus* culture

Three experimental *D. pteronyssinus* cultures (350 g per culture) were maintained in LETI Pharma (Madrid, Spain) facilities for 27–34 days. The inoculum for the test cultures (175 g) was taken from our *D. pteronyssinus* stock cultures. The same batch of the culture medium was used in the three cultures. Temperature and humidity were continuously monitored. The purity and richness of bodies were analyzed before the final harvesting. Then, cultures were inactivated freezing below – 20 °C for at least 48 h.

### Growth and allergens characterization

Seven grams of culture were sampled at different times (day 0, day 7, day 14, day 21 and the final day (27–34)). A general experimental design is indicated in Supplementary Fig. 1. In each case, 10 mg of culture were weighed and resuspended in purified water to quantify mites twice by two skilled people using stereo microscope. The population density was defined as mite number/mg of culture. Additionally, 5 g of each sample from DPT cultures were extracted (1:10) in PBS 0.01M–NaCl 0.15M for 4 h at 2–8 °C, under continuous magnetic stirring. Then, samples were centrifuged at 7300*g* for 30 min, and finally the supernatants were recovered and analyzed by different methods to study the culture evolution:

- Protein content

Total protein content was measured using the Bradford method (Thermo Fisher Scientific, Waltham, MA, USA) according to the manufacturer’s instructions. To determine the protein profile evolution, SDS–PAGE was performed using the 4–12% NuPAGE^®^ Bis-Tris gel system (Invitrogen Corporation, Waltham, MA, USA) under reducing conditions. The maximum volume (40 μl) allowed by the system was loaded in each lane. Finally, gels were stained with Coomassie-Blue R-250 (Bio-Rad, Hercules, CA, USA).

- Allergen quantification

Major allergens (Der p 1, Der p 2 and Der p 23) levels were quantified by ELISA kits (Indoor Biotechnologies, Charlottesville, VA, USA) following manufacturer’s protocol.

- Allergen profile

Allergen profile evolution was studied by western blot. The SDS-PAGE gels were transferred to a Trans-Blot^®^Turbo™ Transfer system (Bio-Rad). Then, the membrane was incubated overnight with a pool of sera from patients allergic to *D. pteronyssinus* (Plasmalab, Everett, WA, USA) and incubated with mouse α-human IgE Fc-HRP (Southern Biotech, Birmingham, AL, USA). Finally, the reaction was developed using a Clarity™ Western ECL substrate (Bio-Rad).

Statistical analyses were performed using GraphPad Prism 9.1.0 software. Normality test was performed, and Pearson r order correlation was used for correlation analysis among the different variables measured.

### Microbiome analysis

For DNA extraction, six hundreds of adult mites were isolated and cleaned from the final cultures. Samples were cleaned by washing twice with 96% ethanol. The surface-cleaned mites were homogenized using a plastic pestle in PBS. The homogenate was extracted using a Genomic DNA NucleoSpin Plant II kit (Macherey-Nagel, Allentown, PA, USA) following manufacturer´s instructions with some minor modifications. The isolated DNA was quantified by Nanodrop and stored at – 20 °C until analysis. To identify bacteria the V3V4 domain of the 16 rRNA gene was amplified (Forward primer: CCTACGGGNGGCWGCAG; Reverse primer: GACTACHVGGGTATCTAATCC). Sequencing was performed on a MiSeq platform (Illumina, San Diego, CA, USA) in the Genomics and NGS Core Facility at Centro de Biología Molecular Severo Ochoa (CBMSO, CSIC-UAM; Madrid, Spain). Quality analyses were performed over reads using FastQC^2^ software. Primer sequences were removed, and not joined and chimeric sequences were discarded using PANDAseq Assembler and QIIME 2 software. More than 80% of joined reads was recovery in every samples. Raw data were deposited in European Nucleotide Archive (Submission ERA10767708). Alignment, construction of the phylogenetic trees, taxonomic assignment analyses and functional profiles were performed using two different platforms, Qiime2 and STAMP.

### Endotoxin content

A hundred mg of each culture was extracted into 4 ml endotoxin-free water overnight and centrifuged. The supernatants were collected and the LPS content was determined according to a Limulus Amebocyte Lysate (LAL) colorimetric kinetic method and based on the methodology approved by European Pharmacopoeia (Council of Europe. European Pharmacopoeia 9.0. Bacterial endotoxins. 01/2018:20614). Briefly, mite culture extract samples were prepared at 1 mg/ml in endotoxin-free ultra-pure water, diluted 1:100 and analyzed in an Endosafe^®^ nexgen-MCSTM system (Charles River, Wilmington, MA, USA) according to manufacturer’s instructions.

## Supplementary Information

Supplementary Figures.

## Data Availability

The dataset Illumina paired ends raw reads (FASTQ) generated during the current study are available in The European Nucleotide Archive (ENA; http://www.ebi.ac.uk/ena/) repository under the study accession number PRJEB51846. The type accession alias are: Culture 1: SAMPLE ERS11206638 A acaro_S95, Culture 2: SAMPLE ERS11206639 B acaro_S96, Culture 3: SAMPLE ERS11206640 C acaro_S97. The rest of data of this study are available from the corresponding author, upon reasonable request.
