# Supplementary material for: Growth, allergen profile and microbiome studies in Dermatophagoides pteronyssinus cultures
Source: Sci Rep. 2023 Jun 30;13:10633. doi: 10.1038/s41598-023-37045-9 (PMC10313659; doi:10.1038/s41598-023-37045-9)
Supplement: Supplementary file 1 — Supplementary Figures. [file 41598_2023_37045_MOESM1_ESM.pdf]

Growth, allergen profile and microbiome studies in *Dermatophagoides pteronyssinus* cultures

Calzada D[1], Martín-López L[1], Carnés J[1].

1. **R&D Unit. Allergy & Immunology. LETI Pharma S.L.U. Madrid, Spain**

Additional information

- **Supplementary Figure 1. Experimental design of the study**

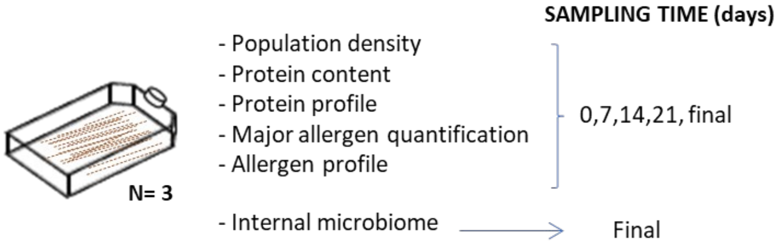

- **Figure 2. Raw and full-length gels and blots. Not editable images.**

Culture 1 Culture 2 Culture 3

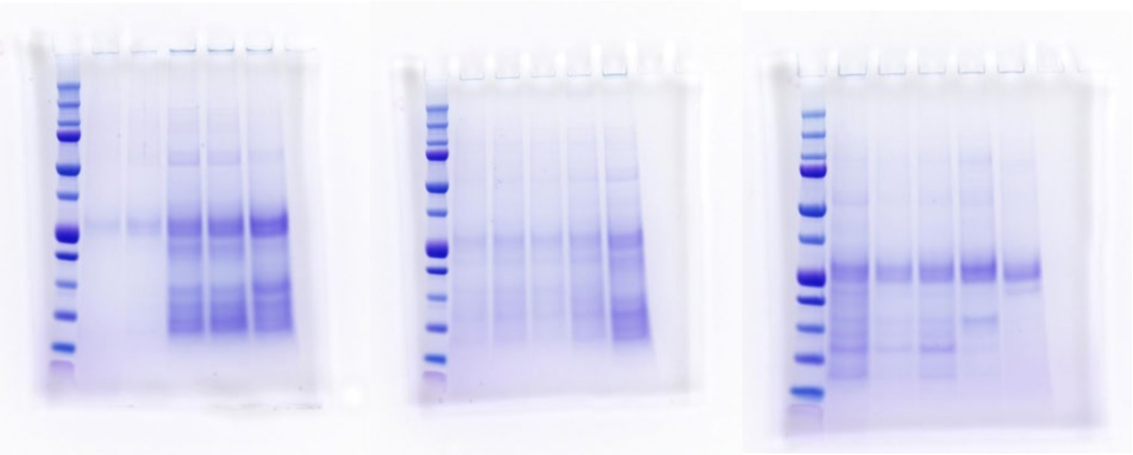

Culture 1 Culture 2 Culture 3

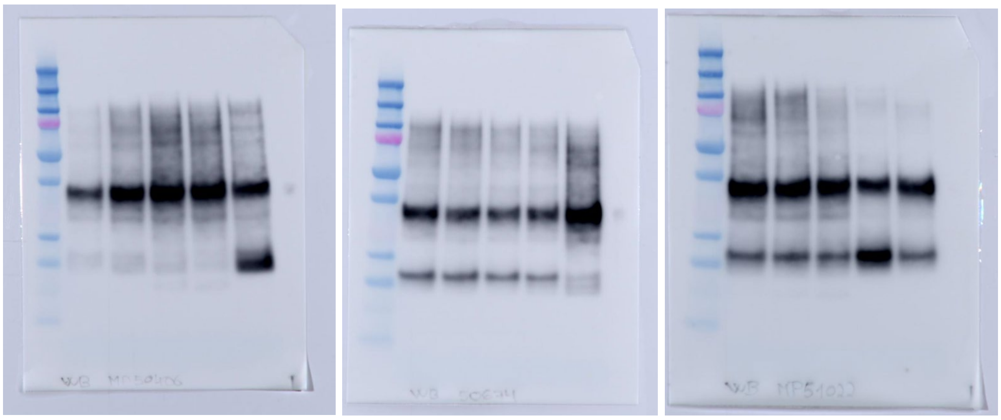

Time of exposure of each bot was 1.5 seconds
